# Comparative analysis of extracellular vesicle isolation methods from human AML bone marrow cells and AML cell lines

**DOI:** 10.3389/fonc.2022.949261

**Published:** 2022-10-03

**Authors:** Jonas B. Lang, Michèle C. Buck, Jennifer Rivière, Oumaima Stambouli, Ken Sachenbacher, Purva Choudhary, Hendrik Dietz, Bernd Giebel, Florian Bassermann, Robert A. J. Oostendorp, Katharina S. Götze, Judith S. Hecker

**Affiliations:** ^1^ Department of Medicine III, Technical University of Munich (TUM), Klinikum rechts der Isar, Munich, Germany; ^2^ Institute for Transfusion Medicine, University Hospital Essen, Essen, Germany; ^3^ Department of Physics, Technical University of Munich (TUM), Munich, Germany; ^4^ Munich Institute of Biomedical Engineering, Technical University of Munich (TUM), Munich, Germany; ^5^ TranslaTUM, Center for Translational Cancer Research, Technical University of Munich (TUM), Munich, Germany

**Keywords:** extracellular vesicles, intercellular communication, AML, bone marrow niche, EV isolation methods

## Abstract

Cellular crosstalk between hematopoietic stem/progenitor cells and the bone marrow (BM) niche is vital for the development and maintenance of myeloid malignancies. These compartments can communicate *via* bidirectional transfer of extracellular vesicles (EVs). EV trafficking in acute myeloid leukemia (AML) plays a crucial role in shaping the BM microenvironment into a leukemia-permissive niche. Although several EV isolation methods have been developed, it remains a major challenge to define the most accurate and reliable procedure. Here, we tested the efficacy and functional assay compatibility of four different EV isolation methods in leukemia-derived EVs: (1) membrane affinity-based: exoEasy Kit alone and (2) in combination with Amicon filtration; (3) precipitation: ExoQuick-TC; and (4) ultracentrifugation (UC). Western blot analysis of EV fractions showed the highest enrichment of EV marker expression (e.g., CD63, HSP70, and TSG101) by precipitation with removal of overabundant soluble proteins [e.g., bovine serum albumin (BSA)], which were not discarded using UC. Besides the presence of damaged EVs after UC, intact EVs were successfully isolated with all methods as evidenced by highly maintained spherical- and cup-shaped vesicles in transmission electron microscopy. Nanoparticle tracking analysis of EV particle size and concentration revealed significant differences in EV isolation efficacy, with exoEasy Kit providing the highest EV yield recovery. Of note, functional assays with exoEasy Kit-isolated EVs showed significant toxicity towards treated target cells [e.g., mesenchymal stromal cells (MSCs)], which was abrogated when combining exoEasy Kit with Amicon filtration. Additionally, MSC treated with green fluorescent protein (GFP)-tagged exoEasy Kit-isolated EVs did not show any EV uptake, while EV isolation by precipitation demonstrated efficient EV internalization. Taken together, the choice of EV isolation procedure significantly impacts the yield and potential functionality of leukemia-derived EVs. The cheapest method (UC) resulted in contaminated and destructed EV fractions, while the isolation method with the highest EV yield (exoEasy Kit) appeared to be incompatible with functional assays. We identified two methods (precipitation-based ExoQuick-TC and membrane affinity-based exoEasy Kit combined with Amicon filtration) yielding pure and intact EVs, also suitable for application in functional assays. This study highlights the importance of selecting the right EV isolation method depending on the desired experimental design.

## 1 Introduction

Acute myeloid leukemia (AML) is a malignant disease characterized by uncontrolled proliferation of non-functional and abnormally differentiated hematopoietic blasts, which accumulate within the bone marrow (BM) and peripheral blood. This proliferation is driven by the transformation of hematopoietic stem and progenitor cells (HSPCs) and rapidly results in BM failure and death if untreated (1). Intensive genetic investigations have started to unravel the complex heterogeneity of AML, which allowed the emergence of new classifications, risk stratifications, predictive biomarkers, and novel therapeutic targets (2–4). However, despite substantial improvements in the treatment landscape of AML, the prognosis and outcome among elderly AML patients are still particularly poor (1, 5). In consequence, it is suggested that targeting intrinsic properties of AML blasts might not be sufficient, and extrinsic factors should be considered. Along this line, targeting the crosstalk between leukemic cells and their BM microenvironment is of special interest, since the BM niche is increasingly recognized as an essential player in the development and maintenance of myeloid malignancies (6, 7). The BM niche comprises various cellular constituents, including HSPC progeny (e.g., megakaryocytes, T cells) and cells of mesenchymal origin [osteoblasts and adipocytes along with their progenitor cells, mesenchymal stromal cells (MSC)], which are important for proliferation, differentiation, and survival of HSPC (8, 9). Previous studies have suggested that leukemic cells are capable of inducing alterations of BM niche function towards a tumor-supporting microenvironment with disruption of normal hematopoiesis (10). In return, this AML-transformed BM niche protects AML cells, leading to clonal persistence and chemo-resistance (7, 11, 12). This leukemia-induced transformation is conducted not only *via* direct cell contact and exchange of soluble factors but also by other crucial mediators such as extracellular vesicles (EVs) (13–15).

EVs are heterogeneous populations of nano- to micro-sized membrane vesicles (30–10,000 nm) with a spherical structure released by essentially all cell types. They have emerged as essential players of intercellular communication, mainly through transfer of their bioactive cargo to recipient cells, including DNA, RNA, lipids, and proteins such as members of the tetraspanin family and glycoproteins, which are commonly used as vesicle markers in Western blot (WB) and flow cytometry analysis (16–19). Increasing evidence suggests that EVs are involved in physiological processes related to stem cell maintenance (20) and tissue repair (21) and in pathological processes, such as cancer (22, 23) or viral infection (24, 25). Moreover, circulating EV-derived microRNAs (miRNAs) were identified as potential early biomarkers to detect minimal residual disease (MRD) in AML (26, 27). Different types of EVs are typically classified according to their size and biogenesis. Most common is the subdivision into exosomes (30–150 nm), which are of endosomal origin, and microvesicles (50–1,000 nm), which are released through calcium-mediated budding from the plasma membrane. However, there is no existing unified classification, and several groups have identified different vesicle populations, such as apoptotic bodies (>1,000 nm) and large vesicles, e.g., oncosomes (>1,000 nm) (28). The isolation of a specific vesicle fraction is technically challenging, as there is an overlap in size, density, and composition between particular exosomes and microvesicles. Since there are many ways to extract and isolate those vesicles, there is no consensus on the optimal isolation method (29–31). The most commonly used isolation method is ultracentrifugation (UC) alone or in combination with other isolation methods (32). However, there are a vast number of further isolation methods, based on specific EV characteristics, such as size or density (32, 33). For example, exoEasy Kit (Qiagen) depends on membrane-based affinity binding columns, and ExoQuick-TC (System Biosciences) is based on polymer precipitation (30, 34). Consequently, the isolated EV subpopulations might exhibit important structural and functional differences depending on the chosen isolation procedure. The impact of different isolation methods on the resulting isolated subpopulation, amount, and purity of EVs still remains unknown. To standardize the work with vesicles, the International Society of Extracellular Vesicles (ISEV) has published position papers, such as the minimal experimental requirements for definition of EVs and their function (MISEV) (35). However, a better characterization of the vesicle fraction and other EV characteristics obtained *via* different EV isolation methods is still needed to guarantee comparability between research groups. In this study, we tested the efficiency and assessed the functional assay compatibility of four different EV isolation methods in AML samples including human primary AML BM cells and the AML cell line MOLM-13 based on: (1) columns alone (membrane affinity-based isolation: exoEasy Kit, Qiagen), (2) and in combination with filter systems (Amicon filters, Merck), (3) precipitation (ExoQuick-TC, System Bioscience), and (4) UC.

## 2 Materials and methods

### 2.1 Isolation procedure of healthy HSPC, MSC, and AML blasts from primary human bone marrow samples

To isolate healthy HSPCs and healthy MSC, healthy human BM samples were obtained from femoral heads of individuals without known hematologic disease undergoing hip replacement surgery (Dr. Martin Nolde, SANA Klinik, München-Solln, Germany) (**Supplementary Table S1**). BM aspiration samples were collected from patients with newly diagnosed AML undergoing routine diagnostic evaluation (**Supplementary Table S2**). All participants provided written informed consent in accordance with the Declaration of Helsinki after approval by the ethics committee of the Technical University of Munich (TUM 538/16S). Healthy BM was harvested by shredding the marrow of the femoral heads using bone-cutting forceps (36). BM fragments were collected in a tube with phosphate-buffered saline (PBS), and BM cells were extracted mechanically by shaking the BM suspension followed by filtration of the cell suspension with a 70-µm cell strainer. BM mononuclear cells (MNCs) from healthy donors and AML patient aspirates were isolated by density gradient centrifugation (Biocoll, density of 1.077 g/L, Germany), frozen in 10% dimethyl sulfoxide (DMSO) and 90% fetal bovine serum (FBS) and stored in liquid nitrogen. After thawing, HSPCs from healthy donors and AML blasts were obtained by using MACS cell separation kits following the manufacturer’s instructions (CD34 or CD33 MicroBeads human, 130-046-702 or 130-045-501, Miltenyi Biotec). Primary MSCs were obtained from the MNC of healthy BM donors by plastic adherence as previously described (37).

### 2.2 Cell culture conditions for healthy HSPC, MSC, and AML blasts

Primary HSPCs were cultured in Iscove’s modified Dulbecco’s medium (IMDM) (Gibco, Life Technologies, IMDM + Glutamax I) with 20% BIT9500 serum substitute (09500, STEMCELL Technologies), 10 µM 2-mercaptoethanol (Gibco, 2-mercaptoethanol, 31350-010), 4 µl/ml ciprofloxacin (Ciprofloxacin Kabi, J01MA02, Fresenius Kabi Austria GmbH), 100 ng/ml SCF (rh-SCF/c-Kit ligand, 255-SC, R&D Systems), 100 ng/ml FLT3-ligand (rh-FLT3 ligand, 308-FK/CF, R&D Systems), 25 ng/ml TPO (rh-Thrombopoietin/Tpo, 288-TP, R&D Systems), 10 ng/ml IL-3 (rh-IL-3 ligand, 203-IL, R&D Systems), and 10 ng/ml IL-6 (rh-IL-3 ligand, 206-IL, R&D Systems). Human MSCs were cultured in low-glucose alphaMEM medium (1 g/L Glucose, M4526, Sigma-Aldrich) with 20 U/ml penicillin/streptomycin (P/S, Gibco, 15140-122) and 2 mM L-glutamine (Gibco, Life Technologies) at a seeding density of 1,000 cells/cm^2^. Pooled fresh human platelet lysate [10% (v/v); prepared as described in (38)] was added to the cell culture medium. Patient-derived AML cells were cultured in Roswell Park Memorial Institute (RPMI) medium with 10% FBS, 20 U/ml P/S, 10 ng/ml FLT3-ligand, and 20 ng/ml TPO. All primary cells were cultivated at 37°C in a humidified atmosphere with 5% CO_2_.

### 2.3 Cell culture conditions for cell lines

The MOLM-13 cell line (ACC 554) used in this study was established from the peripheral blood of a 20-year-old man with AML FAB M5a at relapse after initial myelodysplastic syndrome, carrying an internal tandem duplication of FLT3 (FLT3-ITD) and the CBL deltaExon8 mutation [purchased and cultured as suggested by the German Collection of Microorganisms and Cell Cultures (DSMZ)]. MOLM-13 cells were seeded at a density of 1×10^6^ cells/ml in RPMI medium (Gibco, Life Technologies) with 10% FBS (Gibco, Life Technologies) and 20 U/ml P/S. The murine embryonic stromal cell line EL08-1D2 (39, 40) was cultured on gelatin-coated flasks in alphaMEM medium [Gibco, MEM Alpha Medium (1×) + Glutamax I] containing 15% FBS, 5% horse serum, 20 U/ml P/S, and 10 µM 2-mercaptoethanol. Seeding density was 1,000 cells/cm^2^. The HEK293T-cell line was purchased at DSMZ and cultured in DMEM with 10% FBS and 20 U/ml P/S. All cell lines were cultivated at 37°C in a humidified atmosphere at 5% CO_2_.

### 2.4 Generation of stable CD63-eGFP-expressing cells

The MOLM-13:CD63eGFP stable cell line was generated by lentiviral-mediated gene transfer of human CD63 cDNA (GenBank accession number CR542096) fused to the N-terminus of enhanced green fluorescent protein (eGFP). The plasmids pCL6-CD63eGFP (31), pCD/NL-BH (41), and pcoPE used (42) were kindly provided by Helmut Hanenberg and generated as previously described (31, 43, 44). After receiving the plasmids on filter papers, each dried plasmid was transferred in 100 µl buffer TE (Qiagen) and mixed. After 5 min, 10 µl of the supernatant was used for transformation of *Escherichia coli* (*E. coli*) bacteria: therefore, the mixture of plasmid DNA and bacteria was incubated 30 min on ice, 50 s at 42°C, and additionally 3 min on ice. After that, 250 µl of LB medium (aqua dest. with 10 g/L tryptone, 5 g/L sodium chloride, and 5 g/L yeast extract) was added, and bacterial suspension was incubated overnight at 37°C. Transformed *E. coli* were then selected on an ampicillin-containing LB agar plate. To amplify the plasmid DNA, a single generated *E. coli* colony was harvested and seeded in 5 ml LB medium with 100 µg/ml ampicillin and incubated overnight at 37°C. Plasmid DNA was isolated with the QIAprep Spin Miniprep Kit (Qiagen, 27104) according to the manufacturer and amplified using the CD63 reverse 5′ AGTATCAGAAGTGGCTACGAGGTGAGAATTCATCGGT 3′ and CD63 forward 5′ ACCGATCTCAGCAATGGCGGTGGAAGGAGGAATG 3′ primer. After confirmation of the successful transformation by PCR analysis, the plasmid DNA was isolated using the pegGold Xchange Plasmid Maxi Kit (VWR peqlab, 12-7404-01) according to the manufacturer’s instructions. To produce a lentiviral supernatant, 1×10^6^ HEK293T cells were seeded in a six-well plate, co-transfected with the indicated plasmids by adding a transfection mix (dissolve 5 µg DNA of each plasmid in a total volume of 175 µl aqua dest., add 25 µl 2M CaCl_2_ and 200 µl Hepes 2×) and incubated overnight at 37°C. The next day, the cell medium was exchanged, and the supernatant was collected after an additional 20 h. MOLM-13 cells were transduced by overnight exposure to the viral supernatant, passaged at least three times, and subsequently purified *via* fluorescent cell sorting of GFP^+^ cells using a Fluorescence-Activated Cell Sorting (FACS) Aria III cell sorter (BD Biosciences).

### 2.5 EV production and isolation

#### 2.5.1 Cellular EV production and storage

**Figure 1:**
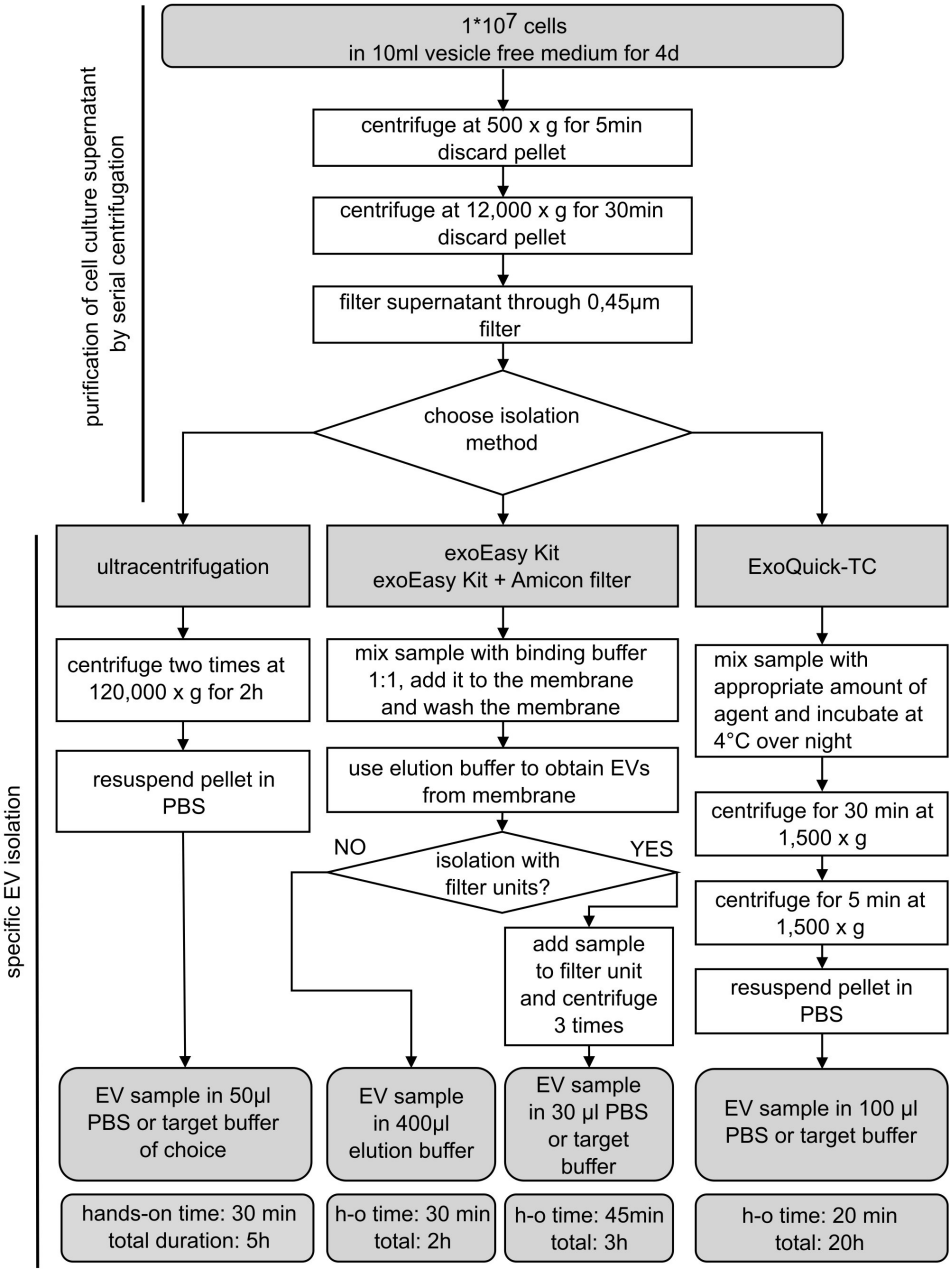
Schematic overview of EV isolation procedures. Extracellular vesicle (EV) isolation from MOLM-13 cells and human AML cells using different commonly used EV isolation methods. Cells were cultured for 4 days in vesicle-free FBS prior to harvesting conditioned medium. Samples were purified by two centrifugation steps and filtering through a 0.45-µm membrane. The purified supernatant was processed by four different isolation methods as depicted in the flowchart. d, days; h, hours; h-o, hands-on; min, minutes.

Samples selected for EV isolation were seeded in an unconditioned, vesicle-free medium: culture medium for MOLM-13 and patient-derived AML cells containing normal FBS was exchanged for a vesicle-free FBS (Gibco, FBS exosome depleted, A2720803)-based medium. For EV production, 1×10^7^ cells were cultured in 10 ml for 4 days. To start the EV isolation process, cells were centrifuged at 500×*g* for 5 min and discarded, while the supernatant was kept as conditioned medium. The conditioned medium was purified by removing dead cells and cell debris with an additional centrifugation step at 12,000×*g* for 30 min followed by filtration through a 0.45-µm filter membrane. After that, EVs were isolated with four different isolation methods based on: (1) a membrane affinity-based procedure alone (exoEasy Kit, Qiagen), (2) and in combination with filter systems (Amicon filters, Merck), (3) precipitation (ExoQuick-TC, System Bioscience), or (4) UC (**Figure 1**). After the specific isolation method, isolated EVs were used immediately or stored at −20°C.

#### 2.5.2 EV isolation with exoEasy Kit (Qiagen) alone and in combination with Amicon filtration

The membrane affinity-based EV isolation procedure was performed with a spin-column system (exoEasyMaxi Kit, 76064, Qiagen). The purified supernatant was mixed with binding buffer (included in the exoEasy Kit from Qiagen and the following buffers) and placed on top of the membrane spin column (34). The sample was centrifuged at 500×*g* for 5 min to capture the vesicles in the membrane and to get rid of spare fluid and proteins. The bound vesicle fraction was cleaned after the addition of washing buffer (Qiagen) to the membrane and additional centrifugation at 5,000×*g* for 5 min. After exchanging the collection tube, the EV fraction was eluted after the addition of an elution buffer (Qiagen) to the column, 1 min incubation at room temperature, and centrifugation at 500×*g* for 5 min. A higher EV recovery was obtained by adding the eluate itself again on top of the column, incubating for 1 min at room temperature, and centrifuging at 5,000×*g* for 5 min. Finally, the vesicle fraction was collected in an end volume of 400 µl provided elution buffer (exoEasy Kit EVs) or further processed (exoEasy Kit with Amicon filtration EVs).

To exchange the elution buffer of exoEasy Kit EVs, the Amicon Ultra-0.5 centrifugal filter unit with a 30 kDa exclusion (UFC503096, Merck Millipore) can be used as an additional procedure. Therefore, after placing 400 µl exoEasy Kit elution buffer on top of the filter unit and centrifuging for 10 min at 14,000×*g*, the flow-through was discarded, and PBS (Gibco) (or buffer of choice) was added on top of the filter. As additional washing steps of the EVs, the filter unit was centrifuged again for 10 min at 14,000×*g*. This was repeated two more times. Finally, the EV fraction was recovered by turning the filter device upside down in a clean collection tube and centrifuging for 2 min at 1,000×*g*. This step allows transfer of the remaining buffer (30–50 µl) containing the concentrated EVs from the membrane to the tube.

#### 2.5.3 EV isolation by precipitation

For EV isolation *via* precipitation (ExoQuick-TC, EXOTC50-A1, System Bioscience), the purified 10 ml conditioned medium was mixed thoroughly with 2 ml of the supplied solution and incubated overnight at 4°C. The next morning, the fluid was centrifuged at 1,500×*g* for 30 min in order to pellet the EVs, and consequently, the supernatant was removed. The sample was then centrifuged once more at 1,500×*g* for 5 min to get rid of residual fluid, after which the EV pellet was resuspended in 100 µl of PBS (or buffer of choice).

#### 2.5.4 EV isolation by UC

To isolate vesicles *via* UC, the purified supernatant was precisely distributed on centrifugal tubes at 2.5 ml/tube and centrifuged at 120,000×*g* at 4°C for 2 h in an ultracentrifuge (Beckmann Coulter, Optima L-90K, SW 60 Ti swinging-bucket rotor) (45). The supernatant was discarded very carefully, and each mostly invisible pellet was resolved in 50 µl PBS. To concentrate the EV fraction, the vesicles from one batch were pooled in one centrifugal tube and centrifuged again at 120,000×*g* for 2 h at 4°C. The supernatant was discarded, and the pellet was resuspended in 50 µl PBS (or buffer of choice).

### 2.6 Protein measurement and Western blot

MOLM-13 cells and MOLM-13-derived EVs were lysed using radioimmunoprecipitation assay (RIPA) buffer (Thermo Fisher, 89900) with protease inhibitors (cOmplete Mini, EDTA-free, 11836170001, Roche, Germany). For cell lysates, 5×10^6^ MOLM-13 cells were centrifuged at 500×*g* for 5 min, and the cell pellet was resuspended in 50 µl lysis buffer. The lysis of EVs was dependent on the specific EV isolation method. EVs isolated with exoEasy Kit (with and without Amicon filters) were lysed by mixing lysis buffer 1:1 with 40 µl EVs eluated in PBS or Qiagen elution buffer, respectively. For EVs isolated with ExoQuick-TC or UC, 50 µl lysis buffer was added directly to the vesicle pellet. For a thorough lysis, these samples were vortexed for 30 s, sonicated (30% power, 10 s, 4 cycles), incubated on ice for 20 min, and centrifuged at 13,000×*g* for 20 min at 4°C to remove cell debris. The protein concentration was measured by DC Protein Assay (BioRad, 500-0111) following the manufacturer’s instructions.

For Western blot analysis, 40 µg of protein was separated by a 4%–20% MiniProtean TGX gel (Bio-Rad, 4568084) for 90 min at 120 V and transferred to a polyvinylidene fluoride (PVDF) membrane (Merck, Immobilon Transfer Membrane, IPVH00010) for 1 h at 1,000 mA. The membrane was blocked for 30 min in TBST buffer (aqua dest. with 50 mM Tris, 150 mM sodium chloride, 0.1% Tween 20) supplemented with either 5% skim milk (for BSA and CD63 detection) or 5% BSA (for all other proteins). Overnight incubation was realized with a specific antibody for the intended target: mouse anti-human TSG101 (1:200, Thermo Scientific, MA1-23296); mouse anti-human CD63 (1:500, BioLegend, 353040); mouse anti-human HSP70 (1:2,000, Abcam, ab5439); rabbit anti-BSA (1:2,000, Sigma-Aldrich, SAB4301142); mouse anti-β-actin (1:2,000, Sigma Aldrich, A5441); and mouse anti-cytochrome c (1:500, BD Pharmigen, 556433). The membranes were washed and incubated with the corresponding horseradish peroxidase-conjugated secondary antibody: goat anti-mouse IgG (1:5,000, Sigma-Aldrich, NA931) or goat anti-rabbit IgG (1:5,000, GE Healthcare, NA934V). Chemiluminescence was uncovered by SuperSignal West ECL femto (Thermo Fisher, 34094) and detected by CL-XPosure film (Thermo Fisher, 34090).

### 2.7 Transmission electron microscopy

EV samples were diluted 1:1 in 4% paraformaldehyde (PFA) (Sigma-Aldrich, HT501128) and incubated 15 min on ice. One drop of the EV/PFA sample was placed on a glow-discharged (45 s, 35 mA at EMS K100X plasma cleaner, Electron Microscopy Sciences) formvar-carbon-coated copper grid (FCF400-Cu, Electron Microscopy Sciences) and incubated for 30 min at RT upside down. The excess solution was removed carefully with a filter paper, and the grid was washed three times with 0.5% BSA in PBS (2 min each). Afterwards, the grid was blocked with 5% BSA in PBS for 30 min and washed again three times with 0.5% BSA in PBS. The grid was contrasted with 2% uranyl formate for 2 min and rested for 10 min prior to imaging after removing the excess solution. Images of the EVs were taken with the FEI Tecnai T12 (120 kV; electron detector, Tietz TEMCAM-F416 camera) and were analyzed with ImageJ (version 1.53a).

### 2.8 Nanoparticle tracking analysis

The size and concentration of EVs were measured using the Zetaview Nanoparticle Tracking Analysis (NTA) machine (Particle Metrix, Germany) (46). The samples were diluted to an optimal concentration of 105–1,010 particles/cm³. Average size, total amount of particles, and concentration of particles were measured after three to five cycles of particle trafficking at 11 positions.

### 2.9 Cell viability analysis by Cell viability analysis by FACS

A total of 30,000 MOLM-13 cells/well were plated in a 96-well plate, and 10,000 EL08-1D2 cells/well were plated in a gelatin coated 24-well plate. Cells were treated with 10% exoEasy Kit elution buffer or 10% exoEasy Kit elution buffer after exchange with PBS *via* filter units (Amicon filters, 30 kDa) or 10% PBS as the resuspension buffer of ExoQuick-TC and of UC and incubated for 2 days. After incubation, the cells were harvested by direct cell transfer from the culture plate to FACS tubes for MOLM-13 cells or after Trypsin (Gibco, Life Technologies, 15400-054) detachment for EL08-1D2. For live/dead staining, cells were incubated with Annexin V (1:250, BD Pharmigen, 51-65874x) and propidium iodide (PI, 1:500, Sigma-Aldrich, P4170-100MG) in Annexin V/PI buffer [10mM Hepes (Gibco, 15630-056), 140mM NaCl, and 2.5mM CaCl_2_ in aqua dest.] for 15 min at 4°C. Fluorescence was measured by a Cyan ADP flow cytometer (Beckman Coulter); data were analyzed with FlowJo software (v10.7.1). Double-negative cells for Annexin V and PI were considered as viable cells.

### 2.10 EV FACS

This procedure was adapted from Inglis et al. (47). A total of 6,000 aldehyde/sulfate latex beads (Thermo Scientific, A37304, 4% w/v, 4 µm) were washed in RPMI (250 µl RPMI centrifuged for 10 min at 1,500×*g* at RT) and added to approximately 2×10^9^ GFP^+^ vesicles harvested from MOLM-13:CD63eGPF cells after 4 days incubation in vesicle-free medium and isolated according to the different previously described isolation methods. After adding beads to EVs, the medium was adjusted to an end volume of 400 µl RPMI and incubated overnight at 4°C. The beads were washed again with RPMI and blocked in 5% BSA in PBS for 3 h at 4°C on a shaker. After washing the beads once more, the pellet was resuspended in 400 µl PBS. The fluorescence was measured by a Cyan ADP flow cytometer (Beckman Coulter), and the data were analyzed with FlowJo software.

### 2.11 EV internalization in EL08-1D2 cells, MSC, and HSPC

For EV uptake experiments, EL08-1D2 cells or MSCs were plated in a Chamber Slide System (Lab-Tek, No. 177445) with a seeding density of 10,000 cells/cm^2^, while 1,000 HSPCs were plated on coated polysine slides (Menzel Gläser Polysine Slides, J2800AMNZ, Thermo Scientific). EV derived from MOLM-13 cells (with and without CD63eGFP transduction) were isolated with ExoQuick-TC. Stromal cells and HSPCs were treated with 1×10^9^ GFP^+^ or GFP^−^ EVs/ml overnight, and an additional treatment was performed with 20 µg/ml heparin as EV transfer blocking agent, therefore serving as a negative control (heparin, 5,000 U/ml; L6510, Batch 0303F, Merck). On the next day, cells were washed with PBS, fixed with 4% PFA for 10 min, and permeabilized with 1% BSA and 0.5% Triton X-100 in PBS. To amplify the GFP signal, cells were incubated overnight with a rabbit anti-GFP antibody (1:400, Thermo Fisher, G10362), washed, and further incubated for 1 h with an Alexa Fluor-conjugated goat anti-rabbit antibody (1:1,000, A-11008, Thermo Fisher Scientific4). For confocal analysis, an additional actin cytoskeleton staining was performed with Phalloidin-iFluor 594 Reagent (1:500, ab176757, Abcam), according to the manufacturer’s instructions. Nuclear counterstaining was done with 4′,6-diamidino-2-phenylindole (DAPI) using the ProLong Diamond Antifade Mountant with DAPI (P36962, Invitrogen) as mounting media. Image acquisition was performed using either an epifluorescence microscope (Leica DM RBE microscope with AxioVision software, Carl Zeiss) for assessment of the percentage of GFP-positive cells or a confocal microscope (Leica SP8 Lightning confocal microscope) for internalization assessment by co-localization analysis between GFP signal and actin cytoskeleton. All images were acquired using the same settings and exposure times along the different conditions. A minimum of four pictures and 150 cells were analyzed per condition. Image processing and analysis were done using ImageJ software.

### 2.12 Statistical analysis

For statistical analysis, Kruskal–Wallis followed by Dunn’s multiple comparisons test was performed using GraphPad Prism software (Version 6.01, GraphPad Inc., La Jolla, CA). The significance level was set at *p*<0.05. The significance level is depicted in the figures as *p<0.05, **p<0.01, ***p<0.001, and ****p<0.0001. Data are presented as mean ± SD.

## 3 Results

Different EV isolation methods were compared in order to assess their efficiency, the purity of the recovered EV fraction, and their compatibility with subsequent functional assays. Therefore, conditioned media from MOLM-13 cells were collected and further processed using equal amounts for the different isolation methods [(1) exoEasy Kit; (2) exoEasy Kit with Amicon filter; (3) ExoQuick-TC and (4) UC] (**Figure 1**). The isolated EV fractions were examined by various analyses, specifically Western blot, transmission electron microscopy (TEM), nanoparticle tracking analysis (NTA), protein measurement, and flow cytometry.

### 3.1 Conventional UC leads to disruption of EVs

**Figure 2:**
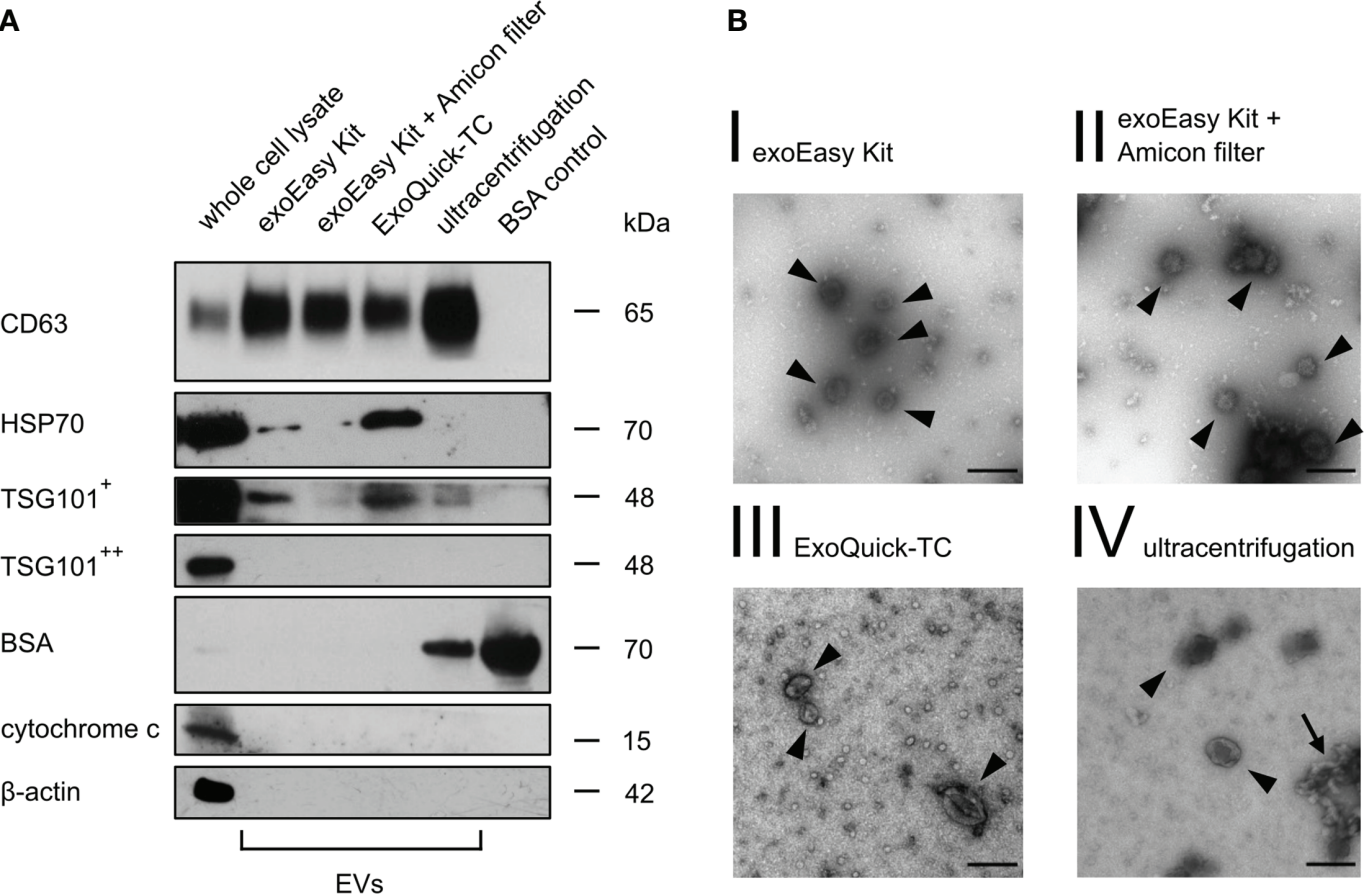
Western blot and transmission electron microscopy of MOLM-13-derived EVs. **(A)** Western blot analysis of MOLM-13 cells and MOLM-13-derived EVs (40 µg/lane) obtained after different isolation methods. As positive vesicle markers CD63, HSP70, and TSG101 were analyzed (^+^ indicates long, ^++^ short exposure time). Cytochrome c and β-actin serve as non-EV markers. BSA (2 µg/lane) was used as purity control for non-EV co-isolating structures. The shown Western blot is a representative figure of n = 3 biological replicates from three independent experiments. **(B)** Transmission electron microscopy of MOLM-13-derived vesicles showed EV-sized cup-shaped structures for every isolation method. Scale bar in the right lower corner was set to 150 nm. The arrowheads display vesicle-like structures, whereas the arrow points out garland-like clotted formations.

In line with MISEV18 recommendations, a transmembrane protein (CD63) and a cytosolic protein (TSG101) regularly enriched in EVs were selected to characterize the different EV fractions in MOLM-13-derived EVs after isolation by Western blot analysis. Both markers, CD63 and TSG101, could be detected using all different isolation methods (**Figure 2A**). HSP70 as an additional cytosolic EV marker was present after isolation with exoEasy Kit and ExoQuick-TC but not after exoEasy Kit with Amicon filter and UC. Considering that EVs share many characteristics, such as density and size, with other structures like proteins or lipoproteins (48, 49), we chose BSA, the most abundant protein in FBS, as a purity control for non-EV co-isolating structures. BSA was not detected after exoEasy Kit, exoEasy Kit with Amicon filter and ExoQuick-TC. In contrast, BSA was present after isolation with UC. In order to detect cellular contaminants, mitochondrial and cytoskeletal proteins (cytochrome c and β-actin) were used as further purity controls, as they are ubiquitously expressed in cells but not enriched in EVs. As expected, both proteins were present in whole cell lysate but not detected in the vesicle fractions.

Furthermore, TEM analysis of MOLM-13-derived EVs showed spherical-shaped structures with a distinct border and a diameter peaking approximately 130 nm, indicating that we were able to isolate an intact vesicle fraction (**Figure 2B**). However, we detected many garland-like clotted structures after UC (**Figure 2B**, IV, arrow), which might indicate aggregation and disruption of EVs by this isolation method, as suggested by different groups (50–52).

### 3.2 EV yield is highly dependent on the isolation method

**Figure 3:**
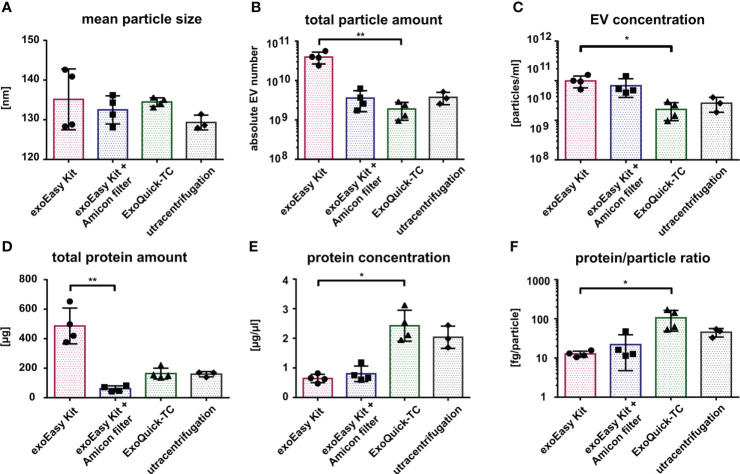
Characterization of MOLM-13-derived EVs according to different isolation methods. Comparison of MOLM-13-derived EVs isolated with different isolation methods using nanoparticle tracking analysis (NTA) **(A–C)** and protein measurement **(D, E)**. Histograms representing the mean particle size **(A)**, total particle amount **(B)**, and particle concentration **(C)** after exoEasy Kit (n = 4), exoEasy Kit + Amicon filtration (n = 4), ExoQuick-TC (n = 4), and UC (n = 3) isolation procedures. Histograms representing total protein amount **(D)** and protein concentration **(E)** after exoEasy Kit (n = 4), exoEasy Kit + Amicon filtration (n = 4), ExoQuick-TC (n = 4), and UC (n = 3) isolation procedures. Histograms representing the protein/particle ratio calculated by dividing the total protein amount by the total particle number from each paired sample **(F)** (n = 3 for UC, n = 4 for other isolation methods). *p<0.05, **p<0.01.

Confirming the results from TEM, nanoparticle tracking analysis (NTA) of MOLM-13-derived vesicles showed no significant differences in EV size between the different isolation methods (**Figure 3A**; **Supplementary Figure S1**) with an average diameter of 133 nm (for exoEasy Kit), 135 nm (for exoEasy Kit with Amicon filter), 134.5 nm (for ExoQuick-TC), and 129.3 nm (for UC). However, NTA revealed major differences in the vesicle amount (**Figure 3B**). The EV yield of 4×10^10^ vesicles was significantly higher after exoEasy Kit isolation compared to ExoQuick-TC (2×10^9^, *p*=0.0094), while a yield of 4×10^9^ vesicles was recovered after isolation with exoEasy Kit with Amicon filter and UC. Comparably high vesicle concentrations were obtained after exoEasy Kit with (1×10^11^ particles/ml) and without Amicon filter (7×10^10^ particles/ml) (**Figure 3C**) and showed a significant decrease upon ExoQuick-TC isolation (2×10^10^ particles/ml, *p*=0.021 compared to exoEasy Kit). UC isolated vesicles showed similar vesicle concentration levels as ExoQuick-TC isolation (3×10^10^ particles/ml).

In accordance with NTA-determined total vesicle amount, the measurement of total protein load (**Figure 3D**) revealed a higher amount after isolation with exoEasy Kit (486 µg) compared to exoEasy Kit with Amicon filter (61 µg, *p*=0.0031), ExoQuick-TC (164 µg), and UC (159.3 µg). However, the protein concentration (**Figure 3E**) differed from our NTA results, as ExoQuick-TC showed the highest protein concentration (2.4 µg/µl), followed by UC (2.0 µg/µl) and exoEasy Kit with Amicon filter (0.8 µg/µl), and is significantly lower after exoEasy Kit isolation (0.6 µg/µl, *p*=0.027).

With the total vesicle amount from the NTA and the total protein amount from the protein measurement, the protein to particle ratio for paired samples was calculated (**Figure 3F**) and revealed that significantly more protein per particle was isolated after ExoQuick-TC (106 µg/particle) compared to exoEasy Kit (13 µg/particle, *p*=0.016). Protein–particle ratio for the exoEasy Kit with Amicon filter was 22 µg/particle and 45 µg/particle for UC.

### 3.3 NTA reveals significant differences in patient-derived AML EVs compared to cell lines

In order to evaluate the effect of the different isolation methods on the EV recovery from more physiological systems, we repeated NTA and protein measurement using primary AML samples (**Supplementary Table S2**).

**Figure 4:**
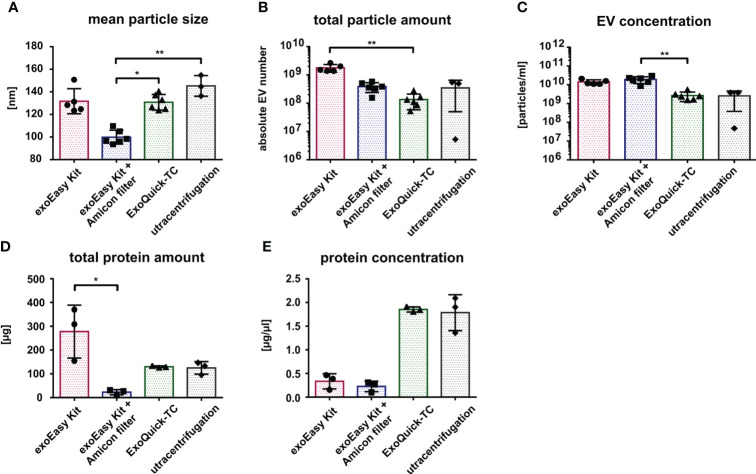
Characterization of primary AML-derived EVs according to different isolation methods. Nanoparticle tracking analysis of size **(A)**, total particle number **(B)**, and EV concentration **(C)** from primary AML-cell-derived EVs isolated by different isolation methods (n = 5 for exoEasy Kit, n = 6 for exoEasy Kit with filter units and ExoQuick-TC, and n = 3 for UC). Measurement of the total protein amount **(D)** and protein concentration **(E)** from primary AML EVs with different isolation methods (n = 3). *p<0.05, **p<0.01.

Similar to what was observed for the MOLM-13-derived EVs, the mean size of AML-derived EVs was 130 nm after isolation with exoEasy Kit and ExoQuick-TC and 145 nm after UC. However, following the exoEasy Kit with Amicon filter isolation procedure, the mean size of the vesicles significantly dropped to 100 nm (**Figure 4A**; *p*=0.047 and *p*=0.0043 for ExoQuick-TC and UC, respectively). Strikingly, the absolute vesicle number after EV isolation from the primary material was 10 times lower compared to MOLM-13 cell line-derived EVs (exoEasy Kit: 2×10^9^; exoEasy Kit with Amicon filter: 4×10^8^; ExoQuick-TC: 2×10^8^; UC: 3×10^8^) (**Figures 3B**, **4B**). However, the differences previously observed between the different isolation methods were the same, with exoEasy Kit yielding the highest amount of total particles (**Figure 4B**). Similar observations were made when comparing EV concentrations from MOLM-13 and primary AML samples (**Figures 3C**, **4C**), since the EV concentration was much lower for primary AML-derived EVs (1.4×10^10^, 1.9×10^10^, 3×10^9^, and 3×10^9^ particles/ml for exoEasy Kit, exoEasy Kit with Amicon filter, ExoQuick-TC, and UC, respectively). In concordance with the cell line-derived EVs, ExoQuick-TC showed again the lowest EV concentration (**Figure 4C**).

Interestingly, despite a 10-fold decrease in total EV amount derived from primary AML samples compared to MOLM-13-derived EVs, the absolute protein amount was only two to three times lower (**Figures 3D**, **4D**). Again, we observed similar variations between the different isolation methods in line with the results from cell line-derived EVs: the exoEasy Kit isolation procedure recovered a higher protein amount (exoEasy Kit: 267 μg; exoEasy Kit with Amicon filter: 23 μg; ExoQuick-TC: 130 μg; UC: 125 μg) as well as a higher protein concentration (exoEasy Kit: 0.3 μg/μl; exoEasy Kit with Amicon filter: 0.2 μg/μl; ExoQuick-TC: 1.9 μg/μl; UC: 1.8 μg/μl) (**Figure 4E**).

### 3.4 Limitations in functional assay compatibility for exoEasy Kit

**Figure 5:**
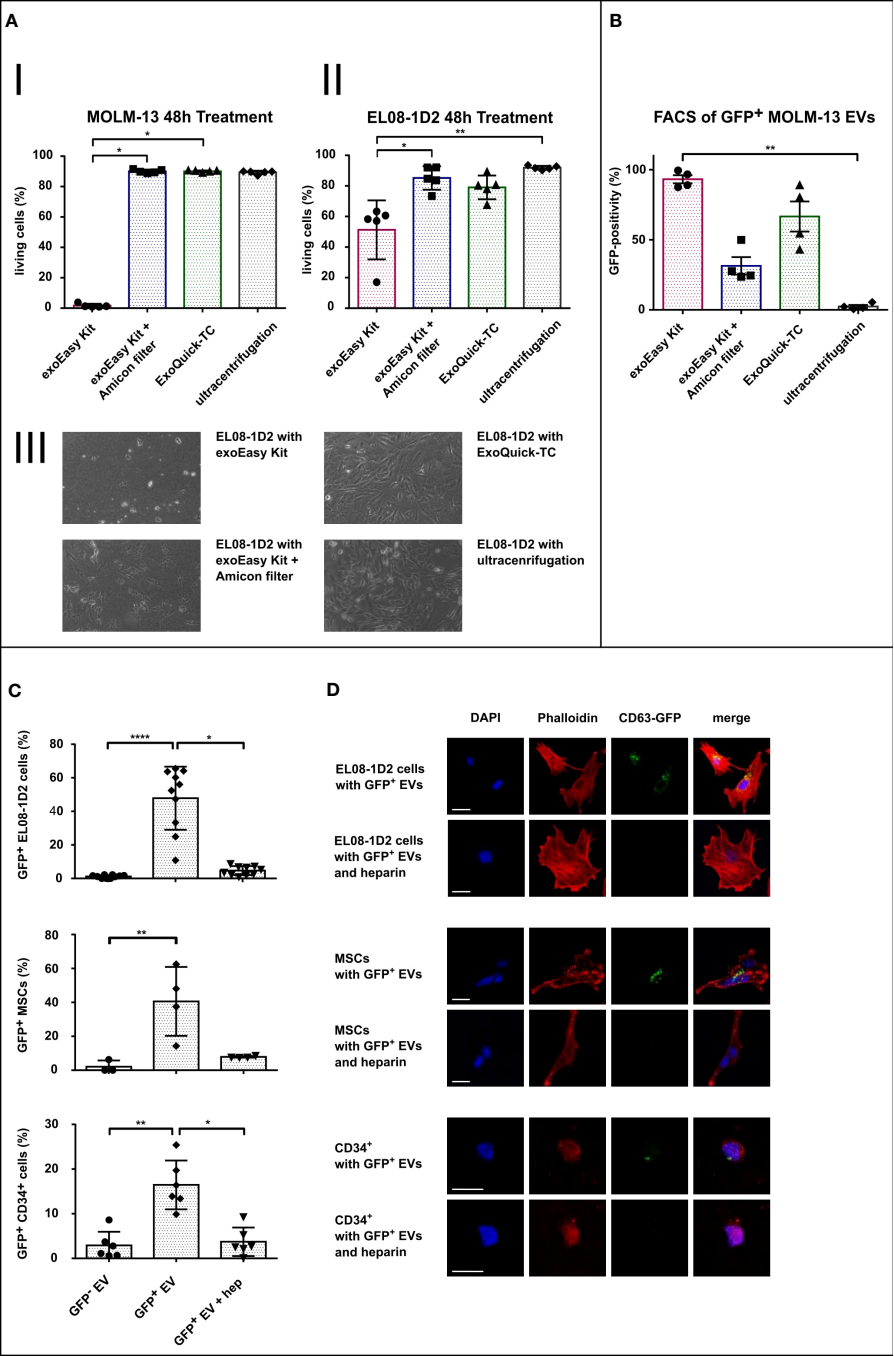
Assessment of EV isolation procedure compatibility with functional assays. **(A)** Histograms representing FACS viability assays of MOLM-13 (I) and EL08-1D2 cells (II) after treatment with different EV isolation buffers for 48 h (n = 5), and representative images of EL08-1D2 cells after 48 h treatment with the different isolation buffers (III). **(B)** FACS analysis of latex beads loaded with CD63-eGFP expressing MOLM-13-derived EVs following different isolation methods (n = 4). **(C, D)** Internalization assay of MOLM-13-derived EV (GFP^-^ EV) or CD63-eGFP expressing MOLM-13-derived EV (GFP^+^ EV) into target cells (EL08-1D2, n = 10 independent experiments; MSC, n = 4 different donors; HSPCs, n = 6 different donors) with or without additional heparin (hep) treatment. Quantification of GFP-positive cells **(C)** and corresponding confocal microscopy images (scale bar set to 20 µm) **(D)**. *p<0.05, **p<0.01, ****p<0.0001.

In order to assess compatibility of EV isolation procedures with functional assays, we first analyzed the effect of the different EV elution buffers on target bone marrow cells. Therefore, suspension (MOLM-13) and adherent stromal cells (EL08-1D2) were treated with 10% of the indicated recovery buffers in cell culture medium (for exoEasy Kit: Qiagen elution buffer; exoEasy Kit with Amicon filter: Qiagen elution buffer replaced by PBS through Amicon filter; ExoQuick-TC: PBS with precipitation agent; UC: PBS), and cell viability was assessed by Annexin V/PI staining. After 48 h, MOLM-13 cells treated with the elution buffer provided in the exoEasy Kit showed a high rate of apoptosis (98.3% ± 1.1%) compared to treatment with other elution buffers (9.9% ± 1.4%, 10.0% ± 0.8%, and 10.7% ± 1.4% for exoEasy Kit with Amicon filter, ExoQuick-TC, and UC respectively) (**Figures 5A–I**; **Supplementary Figure S2**). Similarly, EL08-1D2 cells also revealed a significant increase in apoptosis with detachment of cells after treatment with Qiagen elution buffer (48.7% ± 23.2% apoptotic cells) compared to others (14.8% ± 9.4%, 21.0% ± 10.9%, and 8.1% ± 1.6% for exoEasy Kit with Amicon filter, ExoQuick-TC, and UC, respectively) (**Figure 5A**).

Overall, these results suggest that exoEasy Kit, due to its toxic elution buffer, is incompatible with subsequent functional EV assays on BM cells (both hematopoietic and stromal cells). Nevertheless, the toxic effect of exoEasy Kit could be abrogated by additional filtration (Amicon filters). Furthermore, ExoQuick-TC alone did not alter cellular viability, indicating that with the exception of exoEasy Kit without Amicon filter, all EV isolation methods (exoEasy Kit with Amicon filter, ExoQuick-TC and UC) are of interest for functional assays.

### 3.5 CD63-GFP-positive EVs from different isolation methods exhibit distinct fluorescence patterns

Analyzing effects of EVs in recipient cells assumes a sufficient uptake and internalization of these vesicles, which subsequently leads to the delivery of specific EV cargo inside the target cell. In order to investigate this uptake, techniques for EV visualization have been developed. In particular, the expression of an EV-associated protein (i.e., CD63), in fusion with a fluorescent protein (as GFP), is widely used in the field (31, 53). We thus generated MOLM-13 cells expressing the CD63-eGFP construct and evaluated the impact of the different EV isolation methods on GFP fluorescence of EVs by FACS analysis (**Figure 5B**; **Supplementary Figure S3**). As most EVs are smaller than the wavelength of visible light (380–700 nm), it is challenging to detect them in conventional FACS analysis (54). We therefore loaded EVs on 4-µm large latex beads to capture and visualize those vesicles. Since one latex bead is capable of capturing a total number of up to 3,500 vesicles (55), the resulting fluorescence of each latex bead is the sum fluorescence of many vesicles. The GFP signal of EV-loaded latex beads was analyzed by setting the threshold of positivity using unloaded latex beads (**Supplementary Figure S3A**). Latex beads loaded with exoEasy Kit and ExoQuick-TC isolated EVs showed the highest GFP-positivity (93% and 67%, respectively), whereas smaller fractions of latex beads were GFP-positive when loaded with EVs isolated with exoEasy Kit with Amicon filters (31%) and UC (2%). These results suggest that CD63-eGFP expressing EVs isolated with exoEasy Kit and ExoQuick-TC display better fluorescence properties for downstream internalization experiment analysis.

### 3.6 Cells of the BM microenvironment internalize AML EVs

According to our previous results, the ExoQuick-TC EV isolation method was most suitable for the analysis of EV internalization: besides having no toxic effect on recipient cells, it provided the highest percentage of GFP-positive latex beads. We therefore performed an EV uptake experiment with three different BM target cells: two stromal cell types (the EL08-1D2 cell line and primary human MSC) and primary human HSPC (i.e., CD34^+^ cells) using CD63-eGFP MOLM-13-derived EVs as a proof-of-principle experiment to show compatibility of ExoQuick-TC with further functional assays in different cell types (**Figure 5C**). Since the intrinsic eGFP signal of our vesicles was relatively weak after fixation, we additionally used an anti-GFP antibody for better construct detection. The percentage of GFP-positive cells was quantified *via* fluorescence microscopy (**Figure 5C**). For all three cell types, treatment with CD63-eGFP MOLM-13-derived EVs led to a significant increased percentage of GFP-positivity compared to their respective control treated with MOLM-13-derived EVs not expressing the CD63-eGFP construct (48% *vs*. 1.2%, 40% *vs*. 2%, and 17% *vs*. 3%, respectively, for EL08-1D2, MSC, and CD34^+^ cells). Due to the high GFP-positivity for exoEasy Kit-isolated EVs, we additionally treated EL08-1D2 cells with different concentrations of exoEasy Kit-derived CD63-eGFP MOLM-13 EVs, which did not show any signs of internalization (data not shown).

Since heparin was shown to be a potent EV uptake inhibitor (56), we additionally treated cells with both CD63-eGFP MOLM-13-derived EVs and heparin. We could indeed observe an efficient blocking of EV uptake in all three cell types, assessed by a significant decrease in GFP-positivity with additional heparin treatment (5%, 8%, and 4%, respectively, for EL08-1D2, MSC, and CD34^+^ cells).

In order to confirm that the vesicles were truly internalized and not merely bound to the cell surface, we used a co-localization assay with actin cytoskeleton analyzed by confocal microscopy. Indeed, phalloidin staining revealed GFP-positive vesicles co-localizing with the actin cytoskeleton (yellow pixels in **Figure 5D**), strongly suggesting an actual internalization of EVs by all cell types after 24 h treatment.

## 4 Discussion

Over the last years, there has been an increased interest in examining the characteristics and functional properties of EVs in the context of AML to unveil new therapeutic targets and improve clinical diagnostics (57–59). To perform a valid examination of those vesicles, it is crucial to isolate a pure vesicle fraction by separating them from their co-isolating structures, like lipoproteins or soluble proteins. As the EV field is growing rapidly, there is a constant development of vesicle isolation methods, and many novel isolation kits have recently emerged (60, 61). In this study, we performed a characterization and comparison of AML-derived EVs isolated by four different isolation methods (exoEasy Kit, exoEasy Kit with Amicon filter, ExoQuick-TC, and UC) and investigated their impact on purity and functional assay compatibility according to the MISEV-18 criteria.

In our study, we uncovered that EV isolation using classical UC, despite being widely used in the EV field, was not the most optimal one and suffers from several limitations. The EV characteristics in terms of EV yield and EV or protein concentration after UC isolation were comparable to EVs isolated with ExoQuick-TC, as both methods are based on producing a pelleted EV fraction. Nevertheless, our study in agreement with other groups showed that, apart from a fraction of intact vesicles, TEM analysis revealed EV particle aggregation, suggesting the destruction of a relevant proportion of the isolated EVs (50). This destruction might be due to the high forces with a long turnaround time generated during high-speed centrifugation, which is required for pelleting such small particles, but might damage vesicle membranes. Furthermore, while other EV isolation methods generate pure and intact EV fractions, we and others (31) have shown that UC, in addition to requiring specialized equipment, failed to generate a pure EV fraction, since BSA, a negative EV marker, was co-isolated with EVs. Therefore, the pellet obtained after UC isolation might contain a significant amount of contaminating proteins and debris. Interestingly, several studies showed that using PEG or size-exclusion chromatography in combination with UC results in a purer vesicle fraction (31, 62). Additionally, we observed that UC-isolated EVs from CD63-eGFP MOLM-13 cells lose their GFP-positivity, while treatment of vesicles with polymers such as PEG (which is used to isolate vesicles *via* precipitation similar to ExoQuick-TC) before performing UC reduced the loss of CD63-eGFP fluorescence, also arguing in favor of a possible protection from damage caused by UC (**Supplementary Figure S3B**) (31, 63).

EV isolation using exoEasy Kit alone demonstrated high efficiency in EV recovery, since it resulted in the highest amount and concentration of EVs. These properties indicate the exoEasy Kit to be well suited for subsequent biochemical EV analyses as proteomics or RNA-sequencing, which requires a high amount of material. However, due to the very toxic effect of the EV elution buffer on target cells, this method has its limitations and is unsuitable for functional assays.

Interestingly, while some studies suggest using filtration units to further concentrate the EV solution (64, 65), our data suggest that EV concentration is not increased by this additional step, and EVs are actually lost in large numbers, since we detected a significant decrease in total EV amount *via* NTA. Nevertheless, one important advantage of combining exoEasy Kit with filter units (such as Amicon) is that the toxic elution buffer can be exchanged for a non-toxic buffer, which can then be further used for mechanistic assays. Of note, in GFP-positivity assays of CD63-eGFP MOLM-13-derived EV-loaded latex beads, exoEasy Kit combined with Amicon filters showed very low GFP-positivity compared to the other methods, suggesting a loss of GFP fluorescence, which requires further investigation to unravel the underlying mechanism.

Finally, isolating EVs with ExoQuick-TC recovered the lowest EV amount and concentration but demonstrated no toxicity towards target cells. In addition, GFP-positivity assays of CD63-eGFP MOLM-13-derived EV loaded latex beads after ExoQuick-TC isolation showed higher GFP-positivity compared to exoEasy Kit with Amicon filter isolated EVs. ExoQuick-TC might therefore be a method of choice for downstream functional assays. Indeed, BM niche target cells efficiently internalized ExoQuick-TC isolated EVs, suggesting that additional functional and mechanistic assays on cellular viability, motility, or differentiation can be successfully performed. This is of particular interest for research focusing on the crosstalk between AML cells and their BM niche *via* the secretion of EVs.

Independently of the isolation method used, we noticed that the total amount and concentration of EVs isolated from primary AML material was approximately 10 times lower compared to EVs isolated from AML cell lines. In viability assays prior and after the 4 days of culture in a suitable medium for EV collection, we detected an increased apoptosis rate of primary AML cells compared to MOLM-13 cells, which showed a significantly higher viability at both time points, which might affect EV production and partly explain the lower EV yield from AML samples. Additionally, in EV collection medium, the primary AML cells did not expand, while MOLM-13 cells showed an expansion (data not shown). However, one can also argue that primary AML-cell-derived EVs might be prone to be more delicate and more sensitive to certain isolation procedures than cell lines. In line with this, another noticeable difference is the mean particle size, which was significantly lower after exoEasy Kit with additional filtration for AML-derived EVs compared to all other isolated EVs, and particularly compared to MOLM-13-derived EVs isolated with the same procedure. Hypothetically, this lower EV size in more sensitive, patient-derived EVs might come from a loss of intraluminal fluids, which could occur due to high physical stress during the centrifugation with high centrifugal forces (14,000×*g*) and strong shifts in fluidic amount and concentration of cell culture medium components. However, further studies are required in order to understand the basis of these differences.

In our study, we observed that while EV fractions isolated with different methods revealed similar mean sizes (assessed both in TEM and NTA), we detected important differences in protein to particle ratios and in EV marker expression. In particular, ExoQuick-TC EVs presented the highest enrichment for HSP70 and TSG101 compared to the other methods, suggesting a difference in isolated EV populations. This phenomenon has been described in a previous study by Veerman et al. (29), who showed that different isolation methods (*inter alia* exoEasy Kit and ExoQuick-TC) yielded EV samples of different RNA to particle ratios and protein content. Given this, comparability of results between EV fractions from different isolation methods and different research groups is limited and should be carefully evaluated.

Overall, our data suggest that it is crucial to decide which isolation method is suitable for the specific scientific question being addressed. While for functional assays, it is of utmost importance to work with EV fractions that are *per se* not toxic, other properties can additionally be of interest, such as generating a pure, intact fraction and a high vesicle load in a small volume. For these reasons, exoEasy Kit with additional filtration and ExoQuick-TC represent our methods of choice. However, biochemical analyses of EV cargo (protein and nucleic acid content) usually require a high amount of material, suggesting that exoEasy Kit alone might be the best option, as it provides the highest vesicle amount and total protein amount. Besides the efficiency of the isolation methods and purity of the recovered EV fraction, some practical aspects such as costs (29), hands-on time, and total duration of the isolation procedure (**Figure 1**) should be considered. Regardless of advantages and disadvantages of the different isolation methods, captured EV fractions should be analyzed by NTA, TEM, and Western blot in order to assess EV fraction purity and integrity in a standardized manner.

## Data availability statement

The raw data supporting the conclusions of this article will be made available by the authors, without undue reservation.

## Ethics statement

This study was reviewed and approved by Ethics Committee of the Technical University of Munich (TUM 538/16S) in accordance with the Declaration of Helsinki. The patients/participants provided their written informed consent to participate in this study.

## Author contributions

JL performed experiments, analyzed data, and drafted the manuscript. MB, OS, KS, and PC performed experiments and data analysis. HD, BG, FB, and RO gave conceptual advice and reviewed the manuscript. JR, KG, and JH conceived and supervised the project, analyzed data, and wrote the manuscript. All authors contributed to the article and approved the submitted version.

## Funding

This work was supported by research grants from BMS, the Promotionskolleg of the Else Kröner-Fresenius-Stiftung (2014), and the Deutsche Jose Carreras Leukämiestiftung DJCLS R14/18 (to KG).

## Acknowledgments

We would like to thank the Cell Analysis Core Facility TranslaTUM Munich, Germany; Peter Luppa, Department of Clinical Chemistry, Technical University of Munich (TUM), Klinikum rechts der Isar, Munich, Germany; and Dirk Busch, Department of Microbiology, Technical University of Munich (TUM), Klinikum rechts der Isar, Munich, Germany for technical assistance.

## Conflict of interest

The authors declare that the research was conducted in the absence of any commercial or financial relationships that could be construed as a potential conflict of interest.

## Publisher’s note

All claims expressed in this article are solely those of the authors and do not necessarily represent those of their affiliated organizations, or those of the publisher, the editors and the reviewers. Any product that may be evaluated in this article, or claim that may be made by its manufacturer, is not guaranteed or endorsed by the publisher.
