# Supplementary material for: Comparative analysis of extracellular vesicle isolation methods from human AML bone marrow cells and AML cell lines
Source: Front Oncol. 2022 Oct 3;12:949261. doi: 10.3389/fonc.2022.949261 (PMC9574064; doi:10.3389/fonc.2022.949261)
Supplement: Supplementary file 3 [file DataSheet_1.docx]

Supplementary Material

# Supplementary Materials and Methods

***EV isolation by combined PEG + UC***

To isolate vesicles with polyethylene glycol (PEG) and UC, the purified and filtered conditioned medium was incubated overnight with 10% PEG 6000 (Sigma-Aldrich, Taufkirchen, Germany) at 4°C and distributed on centrifugal tubes at 2.5 ml/tube before being centrifuged at 120 000 x g at 4°C for 2h in an ultracentrifuge (Beckmann Coulter, Optima L-90K, SW 60 Ti swinging-bucket rotor) [(45)](https://paperpile.com/c/sKoFou/BBu7). The supernatant was very carefully discarded, and each mostly invisible pellet was resolved in 50 µl PBS. To concentrate the EV fraction the vesicles from one batch were pooled in one centrifugal tube and centrifuged again at 120 000 x g for 2h at 4°C. Supernatant was discarded and pellet resuspended in 50 µl PBS (or buffer of choice).

# Supplementary Figures


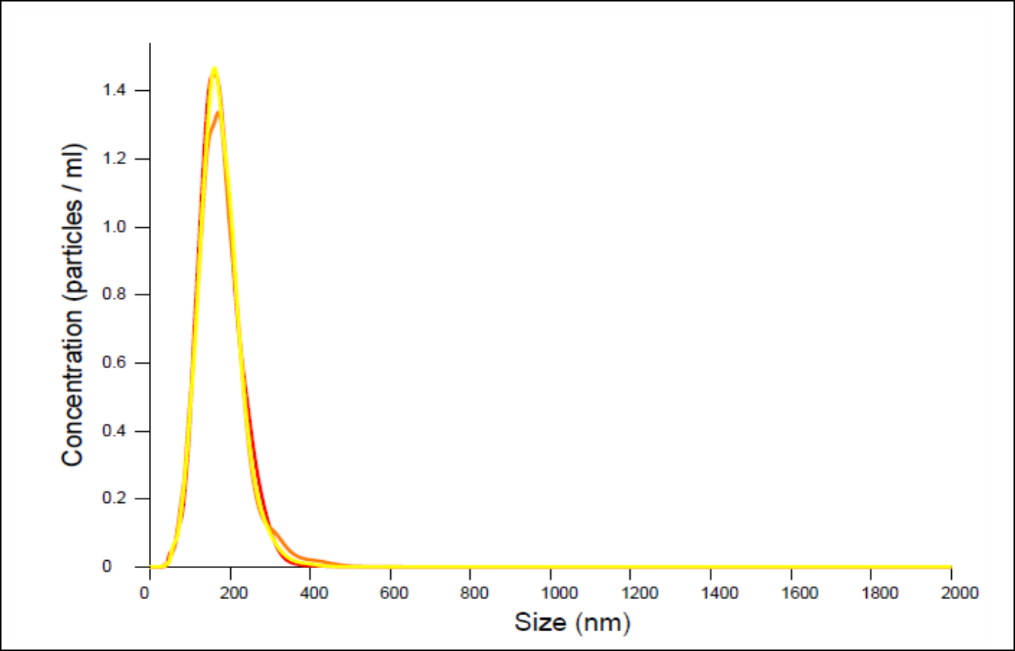


**Supplementary Figure S1.** **Nanoparticle tracking analysis (NTA) of primary AML-derived EVs.** Representative histogram of the particle concentration according to the particle size of isolated EVs analyzed by NTA. The three curves (yellow, orange and red) represent the three technical replicates of an NTA cycle from an AML FLT3-ITD sample. Average concentration and amount were determined afterwards.


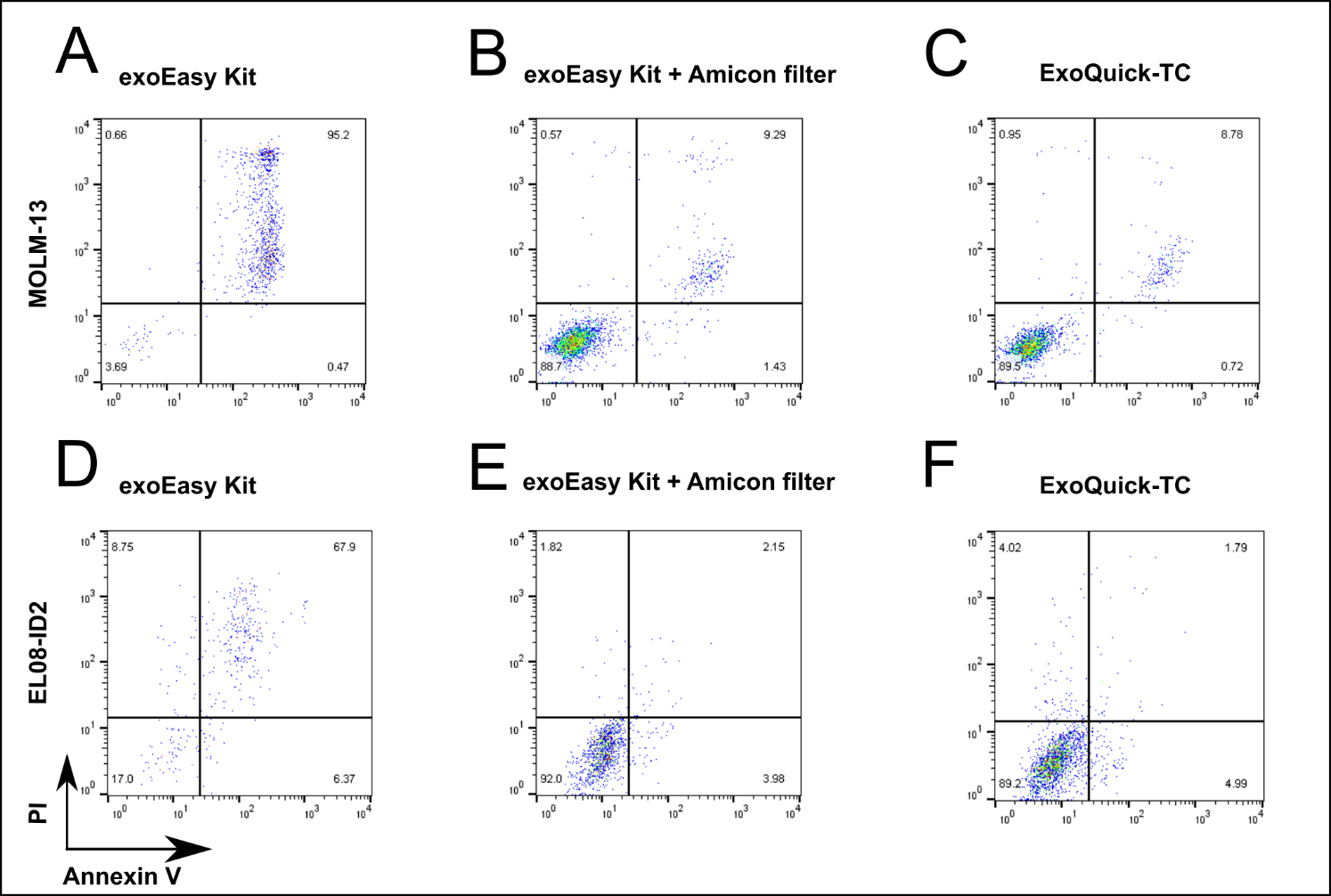


**Supplementary Figure S2.** **Cell viability FACS of MOLM-13 cells and EL08-ID2.** Representative FACS plots of cell viability assay of MOLM-13 cells (**A-C**) and EL08-ID2 cells (**D-F**) performed after 2 days treatment with 10% buffer from the different isolation methods and using AnnexinV/PI staining protocol.


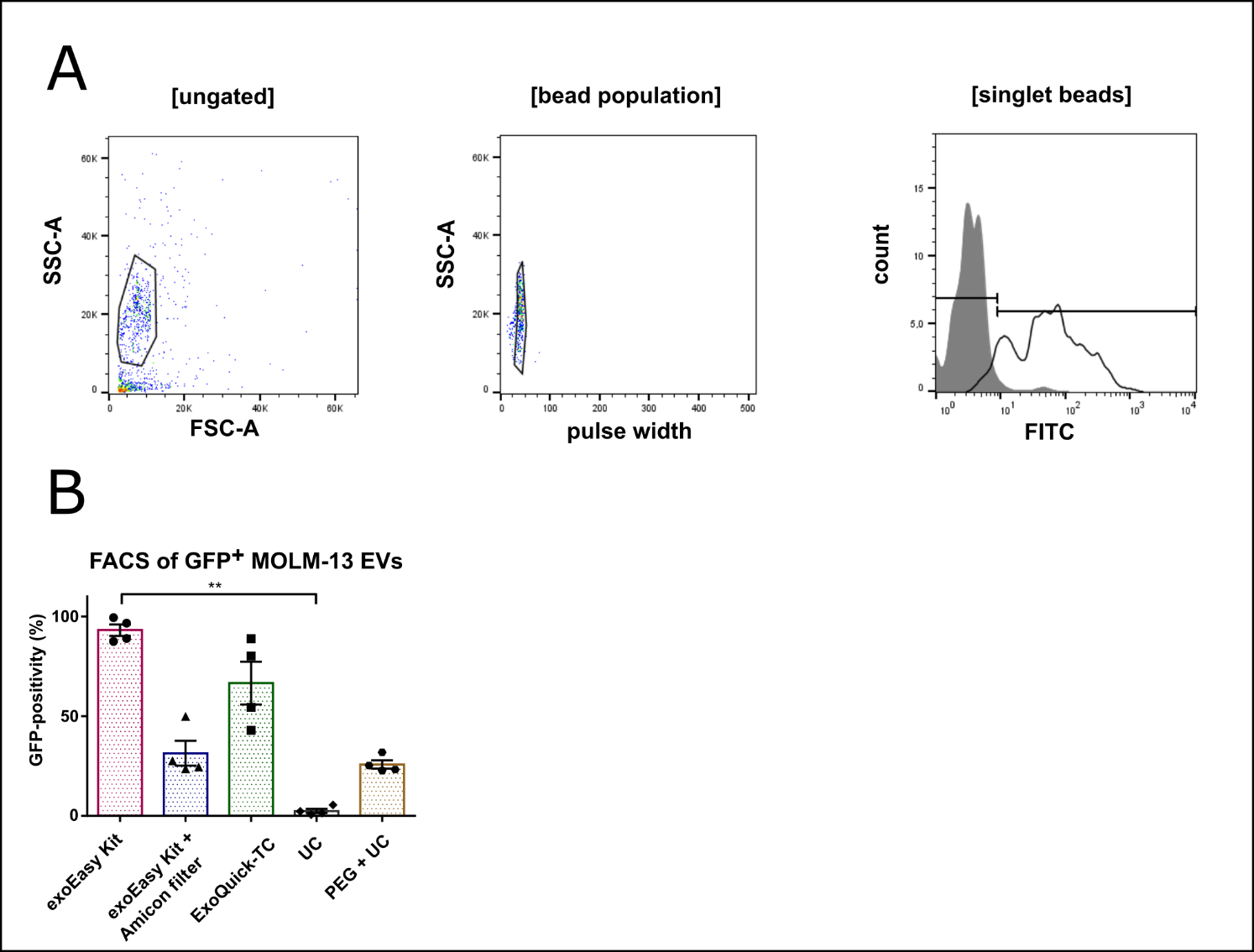


**Supplementary Figure S3.** **FACS analysis of CD63-eGFP expressing MOLM-13-derived EVs loaded on latex beads.** (**A**)**.** FACS gating strategy: after gating the bead population, doublets and triplets were excluded and single bead population was analyzed for GFP fluorescence. Basal beads fluorescence is indicated in gray histogram, black line histogram shows a representative example of latex beads coated with GFP-positive EVs. The threshold for GFP-positivity is set according to the basal bead fluorescence. (**B**). Experiment related to Figure 5B where an additional method was tested (PEG precipitation + UC).

**Abbreviations:** UC: ultracentrifugation, PEG: polyethylene glycol.
